# Early outcomes of the Consolidated Appropriations Act: Missed opportunities for rural dermatology workforce growth

**DOI:** 10.1016/j.jdin.2026.07.001

**Published:** 2026-07-16

**Authors:** Katie Lu, Shari R. Lipner

**Affiliations:** a School of Medicine, University of California, San Francisco, California; b Department of Dermatology, Weill Cornell Medicine, New York, New York

**Keywords:** Consolidated Appropriations Act, graduate medical education, health policy, rural dermatology, rural health care

*To the Editor:* The United States Congress allocated 1000 new residency slots for distribution over 5 years in underserved areas as per the Consolidated Appropriations Act (CAA) of 2021.^1^ Since then, 4 application rounds have been completed, allocating 402 new residency positions based on criteria, including rural designation, Health Professional Shortage Area (HPSA) scores, and institutional capacity (Table I).^2^ Notably, Section 126 of the CAA is one of the only federal funding mechanisms for rural areas that is accessible to specialties such as dermatology.^3^ Therefore, legislative solutions for rural dermatology focus on the CAA.^4^ However, early outcomes demonstrate limited representation of dermatology among awarded positions and a concentration of awards in urban-underserved settings, raising concerns about unmet rural dermatology workforce needs.

**Table I:** Section 126 of the CAA 2021 qualification criteria

Positions must qualify in at least one of the following 4 categories∗^,^†:
(1) Hospitals in rural areas (or treated as being located in a rural area under the law)
(2) Hospitals training a no. of residents in excess of their GME cap
(3) Hospitals in states with new medical schools or branch campuses
(4) Hospitals that serve areas designated as health professional shortage areas

*CAA,* Consolidated Appropriations Act; *FTE*, full-time equivalent; *GME*, Graduate Medical Education.

∗ Additionally, Section 126 requires that at least 10% of the cap slots go to hospitals in each of the 4 categories and that no single hospital can receive more than 25 FTE resident cap slots.

† Section 4122 of the CAA 2023 makes available an additional 200 FTE resident cap slots beginning in fiscal year 2026. At least 100 of the positions made available shall be distributed for psychiatry or psychiatry subspecialty residency training programs.

Publicly available Centers for Medicare & Medicaid Services’ Section 126 awardee data from the first 4 CAA allocation rounds were reviewed. For each specialty, descriptive statistics were performed, including the number of positions awarded, mean HPSA scores, and urban versus rural classification using HPSA classifications (Table II). HPSA scores range from 0 to 25, with higher scores indicating greater workforce shortages.^5^

**Table II:** Distribution of Section 126 GME awards by specialty and setting

Specialty	No. of awards (% of total positions)	Mean HPSA score (SD)	No. in rural or partially rural settings (% of total positions)
Internal medicine (including IM-Pediatrics and subspecialties)	84 (20.9)	16.3 (2.67)	33 (39.3)
Psychiatry (all subspecialties combined)	81 (20.1)	17.4 (2.04)	20 (24.7)
Family medicine	82 (20.4)	16.2 (2.05)	50 (61.0)
Pediatrics (including subspecialties)	28 (6.97)	15.3 (1.78)	8 (28.9)
Surgery (all surgical subspecialties combined)	36 (8.96)	16.6 (2.29)	9 (25.0)
OBGYN (including maternal-fetal medicine)	20 (4.98)	16.7 (2.08)	10 (50.0)
Neurology (including child/pediatric and neuro-intervention)	20 (4.98)	16.1 (2.13)	9 (45.0)
Emergency medicine (including pediatric emergency medicine)	14 (3.48)	16.3 (1.53)	2 (14.3)
Anesthesiology	12 (29.9)	17.9 (2.06)	0 (0)
Radiology (diagnostic + interventional)	8 (1.99)	17.9 (2.32)	2 (25.0)
Urology	5 (1.24)	16.8 (2.32)	2 (40.0)
Addiction medicine	4 (.995)	17 (1.22)	0 (0)
Physical medicine and rehabilitation	3 (.746)	16 (2.16)	1 (33.3)
Dermatology	2 (.498)	20 (0)	1 (50.0)
Transitional year	2 (.498)	15.5 (.50)	1 (50.0)
Ophthalmology	1 (.249)	18 (0)	1 (100)

*GME*, Graduate Medical Education; *HPSA*, health professional shortage area; *IM*, internal medicine; *OBGYN*, obstetrics gynecology.

Internal medicine, family medicine, and psychiatry represented the majority of awards (61.4%). Dermatology accounted for 2 positions (0.5%), the second lowest of all represented specialties. Mean HPSA scores were generally similar across specialties, ranging from 15.3 to 17.9. Overall, only 149 (37.1%) awarded positions were in partially rural or rural areas. One of 2 dermatology positions was in a rural or partially rural institution.

The CAA represents a step forward for rural dermatology access, and evaluation of early outcomes is important for future opportunities to expand growth. However, dermatology represented only <1% of awards across 4 allocation rounds. Additionally, the low number of overall positions awarded to hospitals in rural or partially rural areas indicates that the current criteria may prefer academic institutions with existing Graduate Medical Education infrastructure in urban areas (Table II). Consequently, expanding dermatology positions within existing urban residency programs that already meet CAA eligibility criteria may be a practical strategy. One example is to establish rural track positions at urban institutions, such as the position awarded to the University of Mississippi, where residents spend ∼25% of their time at a rural satellite clinic. Other future policy modifications could include the designation of dermatology as a priority specialty category, similar to the prioritization of psychiatry introduced in Section 4122 of the CAA 2023 (Table I). However, a trade-off is that expanding dermatology positions may reduce other high-priority, specialty residency positions. Dermatologists can support the rural dermatology workforce by supporting new legislation that focuses on rural locations and HPSAs, such as the Resident Physician Shortage Reduction Act.

Early outcomes from the CAA demonstrate that although there is new federal funding available for rural dermatology outreach, current allocation patterns have resulted in minimal dermatology representation and continued concentration of positions within urban institutions. Future efforts to address rural dermatology workforce shortages may require targeted policy prioritization for dermatology, expansion of rural training tracks affiliated with established academic centers, and support of legislation focused on rural and HPSA–based physician workforce development.^3^

### Declaration of generative AI and AI-assisted technologies in the writing process

AI was not used in the preparation of this work.

## Conflicts of interest

Dr Lipner has served as a consultant for Veradermics. Author Lu has no conflicts of interest to declare.
